# *Synotis xinningensis* (Asteraceae), a new species from Hunan, China

**DOI:** 10.1186/1999-3110-54-16

**Published:** 2013-08-23

**Authors:** Ming Tang, Long-Yuan Wang, Qin-Er Yang

**Affiliations:** 1 Key Laboratory of Plant Resources Conservation and Sustainable Utilization, South China Botanical Garden, Chinese Academy of Sciences, Xingke Road, Tianhe District, Guangzhou, 510650 China; 2 School of Life Science, Sun Yatsen University, Guangzhou, 510275 China; 3 University of Chinese Academy of Sciences, Beijing, 100049 China

**Keywords:** Asteraceae, Chromosome number, Karyotype, Senecioneae, *Synotis xinningensis*

## Abstract

**Background:**

*Synotis* is one of the several genera within Senecioneae (Asteraceae) with more than 40 species that are mainly distributed in China or in the Sino-Himalayan region. During a botanical expedition in central and southwestern China in 2011, we found an unusual population of *Synotis* in southwestern Hunan Province. To determine the taxonomic identity of the population, we carried out gross-morphological, floral micromorphological, and cytological observations.

**Results:**

Our gross-morphological observations have shown that the population is most similar to *Synotis changiana* Y. L. Chen, but readily distinguishable in the discoid capitula (vs. radiate), and in the bracts of calyculus 9–10 (vs. 6–8), 6–7 mm long (vs. 3–4 mm). The floral micromorphological observations on the population and *S. changiana* agree with previous reports for other species of *Synotis*. The chromosomes of the population are counted to be 2*n* = 40 + 0–1B. Its karyotype is formulated as 2*n* = 22m + 14sm + 4st.

**Conclusions:**

The population is determined to represent a new species, i.e. *Synotis xinningensis* M. Tang & Q. E. Yang, which is described herein. The new species belongs to *Synotis* ser. *Synotis*.

**Electronic supplementary material:**

The online version of this article (doi:10.1186/1999-3110-54-16) contains supplementary material, which is available to authorized users.

## Background

Within Senecioneae (Asteraceae), there are several genera with more than 40 species that are mainly in China or in the Sino-Himalayan region. They include *Cremanthodium* Benth., *Ligularia* Cass., *Parasenecio* W. W. Smith & Small, *Sinosenecio* B. Nord., and *Synotis* (C. B. Clarke) C. Jeffrey & Y. L. Chen (Jeffrey and Chen 1984; Chen et al. 2011). One of us (Yang) and colleagues have a strong interest in these genera and are carrying out in-depth systematic studies of them. These studies have focused on *Sinosenecio* (e.g., Liu and Yang 2010, 2011a, b, c, d, 2012; Liu et al. 2009, 2010, 2011; Zhang et al. 2008) and have made important contributions to our knowledge of the genus (Nordenstam and Pelser 2011). The genus now under study is *Synotis*.

*Synotis* is a genus of about 54 species, all endemic to the Sino-Himalayan region except for *S. atractylidifolia* (Y. Ling) C. Jeffrey & Y. L. Chen, which occurs in northern China (Jeffrey and Chen 1984; Chen 1999; Nordenstam 2007; Chen et al. 2011). Chen (1999) and Chen et al. (2011) recorded 43 species of *Synotis* in China.

During a botanical expedition in central and southwestern China in 2011, we found an unusual population of *Synotis* on Lang Shan, Xinning County, in southwestern Hunan Province. The plants were most similar to *S. changiana* Y. L. Chen, a species described from Guangxi (Chen 1995), but very readily distinguishable in the discoid capitula. Further examination revealed additional differences in the number and length of the bracts of calyculus. We determined that the population represents an undescribed species, i.e. *Synotis xinningensis* M. Tang & Q. E. Yang, which is here described. In 2012, we found two additional gatherings of the new species in the Herbarium of the Institute of Botany, Chinese Academy of Sciences (PE).

## Methods

### Floral micromorphological character observations

For observation of the filament collar and anther endothecial cell wall thickenings of *Synotis xinningensis* (voucher: *Long-yuan Wang* & *Ming Tang 123*, HAST, IBSC) and its putative closest relative, *S. changiana* (voucher: *Long-yuan Wang* & *Ming Tang 111*, HAST, IBSC), heads were boiled in distilled water for 3 min, and then fixed in Carnoy’s solution (glacial acetic acid: absolute ethanol = 1: 3). Mature disc florets removed from the fixed heads were dehydrated in 70% ethanol for 30 min, then placed in 99% ethanol for 1 h before they were treated with 5% NaOH overnight. The anther tissue was isolated from the florets on the slide, flooded with 50% glycerol and a cover slip was applied. Samples were then examined at 100× (filament collar) and 400× (endothecial cell wall thickenings) magnification by light microscopy and photographed.

### Chromosomal observations

For chromosome observation of *Synotis xinningensis* (voucher: *Long-yuan Wang & Ming Tang 123*, HAST, IBSC) and *S. changiana* (voucher: *Long-yuan Wang & Ming Tang 111*, HAST, IBSC), root tips were pretreated with 0.1% colchicine for 2.5 h before being fixed in Carnoy’s solution (glacial acetic acid: absolute ethanol = 1: 3), then macerated in a 1:1 mixture of 45% acetic acid and 1 M HCl at 37°C for 45 min, stained and squashed in Carbol fuchsin.

## Results and discussion

### Taxonomic treatment

**Figure 1:**
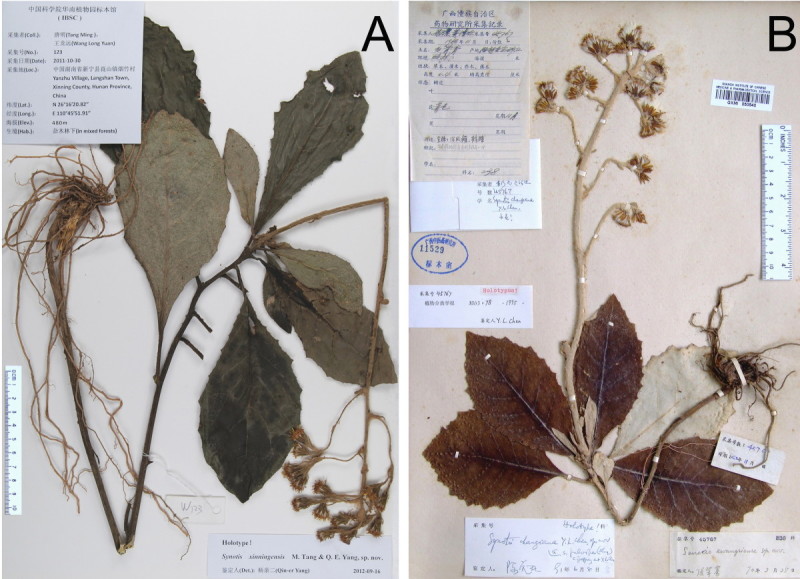
***Synotis xinningensis*** **and** ***S*** **.** ***changiana*** **. A**, *S*. *xinningensis*, China, Hunan, Xinning County, Lang Shan, *Long-yuan Wang* &*Ming Tang 123* (holotype, IBSC); **B**, *S*. *changiana*, China, Guangxi, Yangshuo County, Jinbao, *Nai-kuan Liang* &*Zeng-ren Huang 45767* (wrongly cited as *45765* in the protologue) (holotype, GXMI).

**Figure 2:**
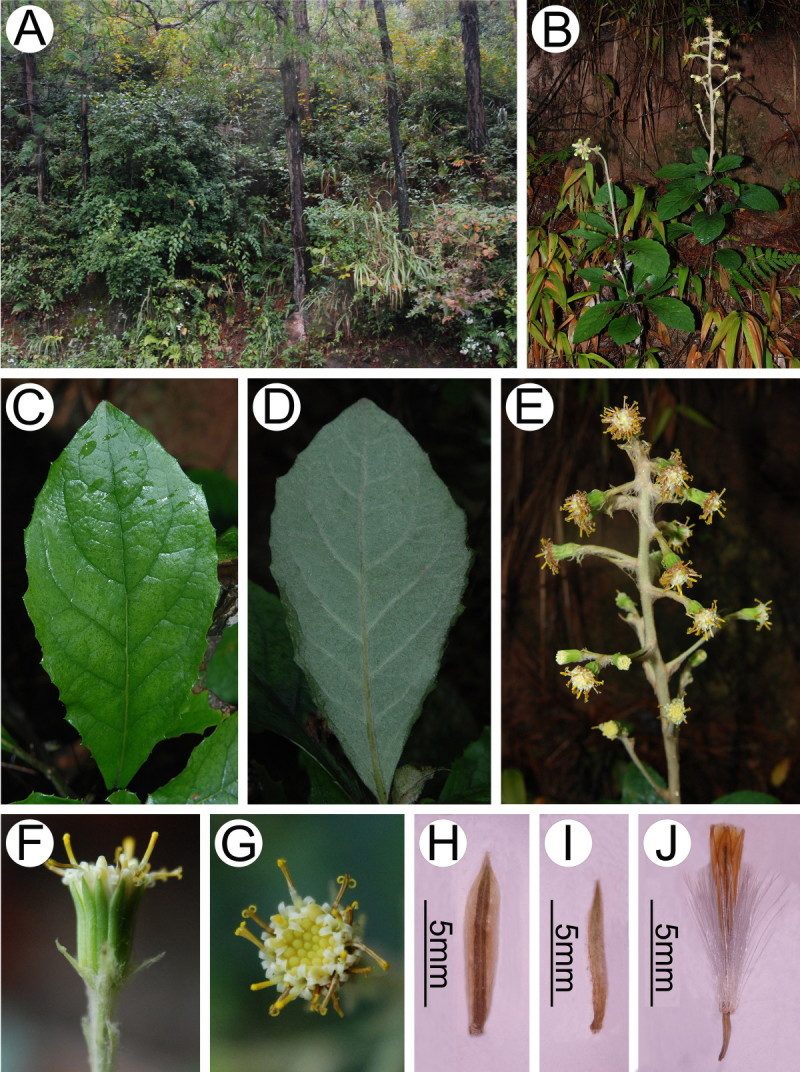
***Synotis xinningensis*** **. A**, Habitat; **B**, Habit; **C**, Leaf blade (adaxial side); **D**, Leaf blade (abaxial side); **E**, Synflorescence; **F**, Capitulum (side view); **G**, Capitulum (top view); **H**, Phyllary (outer side); **I**, Bract of calyculus (outer side); **J**, Disc floret. All from *Long-yuan Wang* &*Ming Tang 123* (HAST, IBSC) from the type locality.

***Synotis xinningensis*** M. Tang & Q. E. Yang, sp. nov.—TYPE: CHINA. Hunan, Xinning County, Lang Shan, alt. ca. 480 m, open mixed forests, 28 Oct 2011, *Long-yuan Wang* & *Ming Tang 123* (holotype, IBSC; isotype, HAST). Figures 1A and 2.

#### Description

Herbs, erect, rhizomatous. Rhizome stout, 0.6–1 cm in diameter. Vegetative stem solitary, 60–85 cm tall, simple, 3–5 mm in diameter at base, lower part naked, at first arachnoid, more or less glabrescent, upper part fulvous tomentose. Leaves usually densely crowded at middle of stem, rosulate or subrosulate, petiolate; petiole 3.5–4.5 cm long, stout, densely tomentose; blade obovate-lanceolate, 8–15 cm long, 4.5–8 cm broad, papery, base cuneate-attenuate, margin irregularly mucronately sinuate-serrate, apex obtuse to subacute, abaxially densely grayish white arachnoid-tomentose, adaxially dark green, arachnoid, glabrescent, pinnately veined, lateral veins 6–7 pairs, arcuate-ascending. Upper leaves sessile, bract-like, linear. Capitula discoid, 4–21 in terminal corymbs; synflorescence to 1–18 cm long, densely fulvous tomentose, subsessile or short-pedunculate, bracteate at base; bracts linear, 10–13 mm long, acute. Involucres campanulate, 8–10 mm long, 8–12 mm broad, base fulvous tomentose, calyculate; bracts of calyculus 9–10, 6–7 mm long, linear-subulate, acute; phyllaries 12–13, oblong-lanceolate, 8–10 mm long, 2–2.5 mm broad, herbaceous, glabrous, margin broadly scarious, inconspicuously 3-veined, apically slightly acute or obtuse. Ray florets absent. Disc florets many; corolla yellow, 9–11 mm long, tube 3–3.5 mm long, limb narrowly funnelform; lobes oblong-lanceolate, acute. Anthers linear, 3.5–4 mm long, basally caudate, antheropodia slightly expanded. Style branches recurved, apically obtuse, papillose. Achenes ca. 2 mm long, glabrous. Pappus white, 6–7 mm long.

#### Additional specimens examined

CHINA. HUNAN: Xinning County, Luoyuan, Jigong Shan, in forests at mountaintop, alt. 1000 m, 9 Aug 1985, *Yi-bo Luo 3060* (PE); Xinning County, Bajiaozhai, in forests on mountain slopes, alt. 450 m, 13 Sept 1985, *Yi-bo Luo 3349* (PE).

#### Etymology

The specific epithet ‘*xinningensis*’ is derived from the type locality, Xinning County, southwestern Hunan Province, China.

#### Phenology

Flowering October; fruiting November.

#### Distribution and habitat

**Figure 3:**
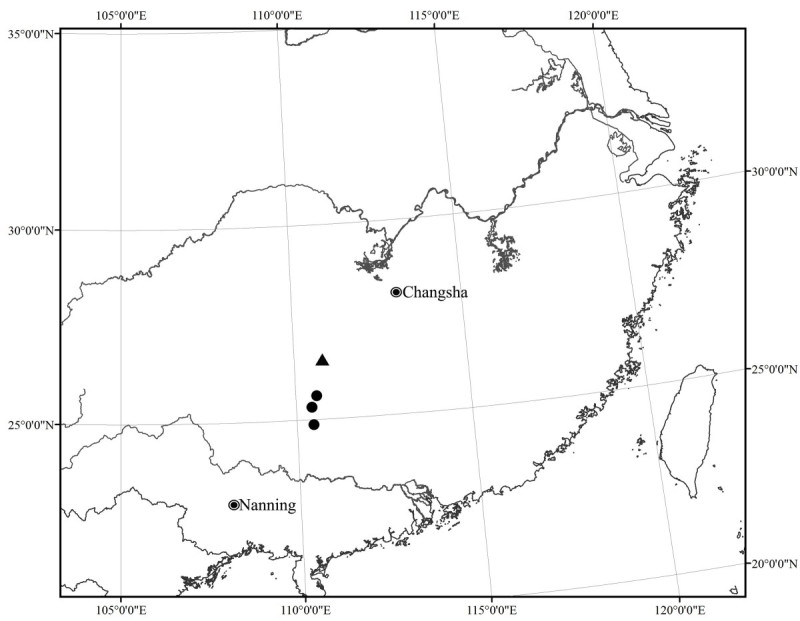
**Distribution of** ***Synotis xinningensis*** **(▲) and** ***S. changiana*** **(●).**

*Synotis xinningensis* is currently known only from Xinning County, southwestern Hunan Province, China (Figure 3). It grows in open mixed forests at 450–1,000 m above sea level.

#### Floral micromorphological characters

**Figure 4:**
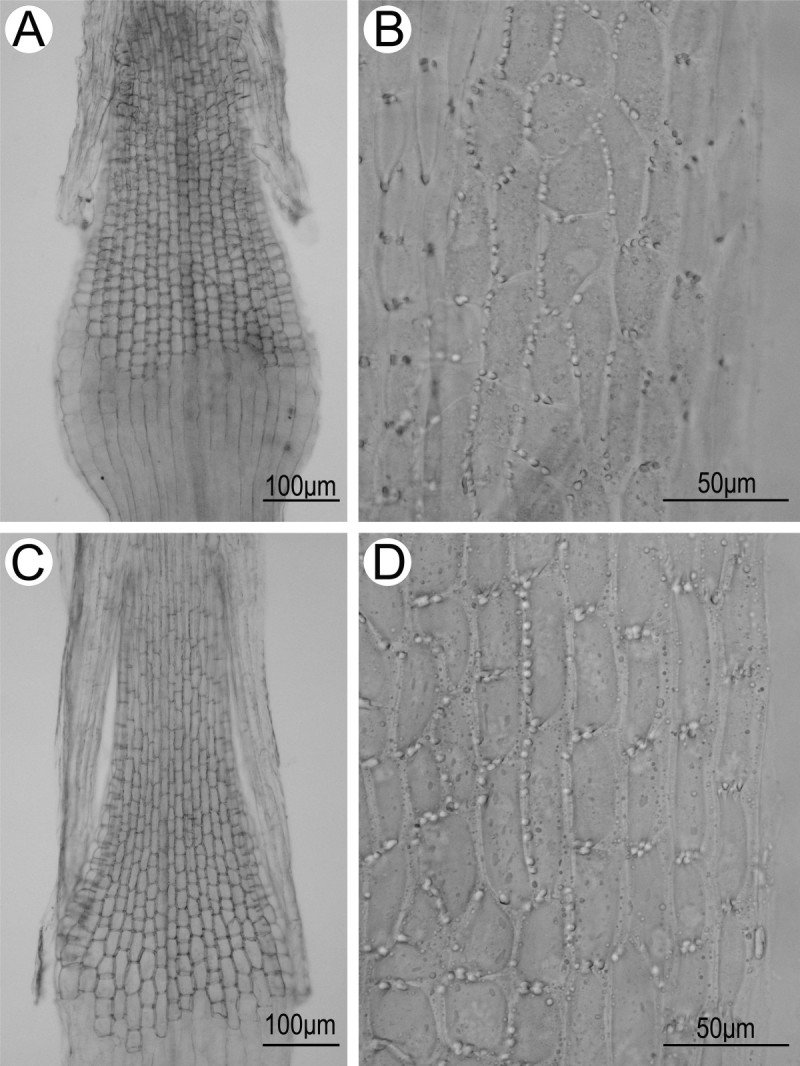
**Filament collar (A, C) and anther endothecial cell wall thickenings (B, D) of** ***Synotis xinningensis*** **(A, B) and** ***S. changiana*** **(C, D).** **A** and **B** from *Long-yuan Wang* &*Ming Tang 123* (HAST, IBSC) from the type locality; C and D from *Long-yuan Wang* &*Ming Tang 111* (HAST, IBSC) from Xing’an County, Guangxi, China.

As shown in Figure 4A, C, the filament collars of both *Synotis xinningensis* and *S. changiana* are balusterform, being basally dilated and consisting of larger cells, conforming to the results reported previously for some other species of *Synotis* (Jeffrey and Chen 1984). The anther endothecial cell wall thickenings of *S. xinningensis* (Figure 4B) were distributed along all the inner walls of the endothecial cells, and thus were radial. *Synotis changiana* also had the same pattern of anther endothecial cell wall thickenings (Figure 4D). The observations agree with previous reports for other species of *Synotis* (Jeffrey and Chen 1984). The floral micromorphological characters indicate that *Synotis* is a member of subtribe Senecioninae as defined by Nordenstam (2007).

#### Chromosome cytology

**Figure 5:**
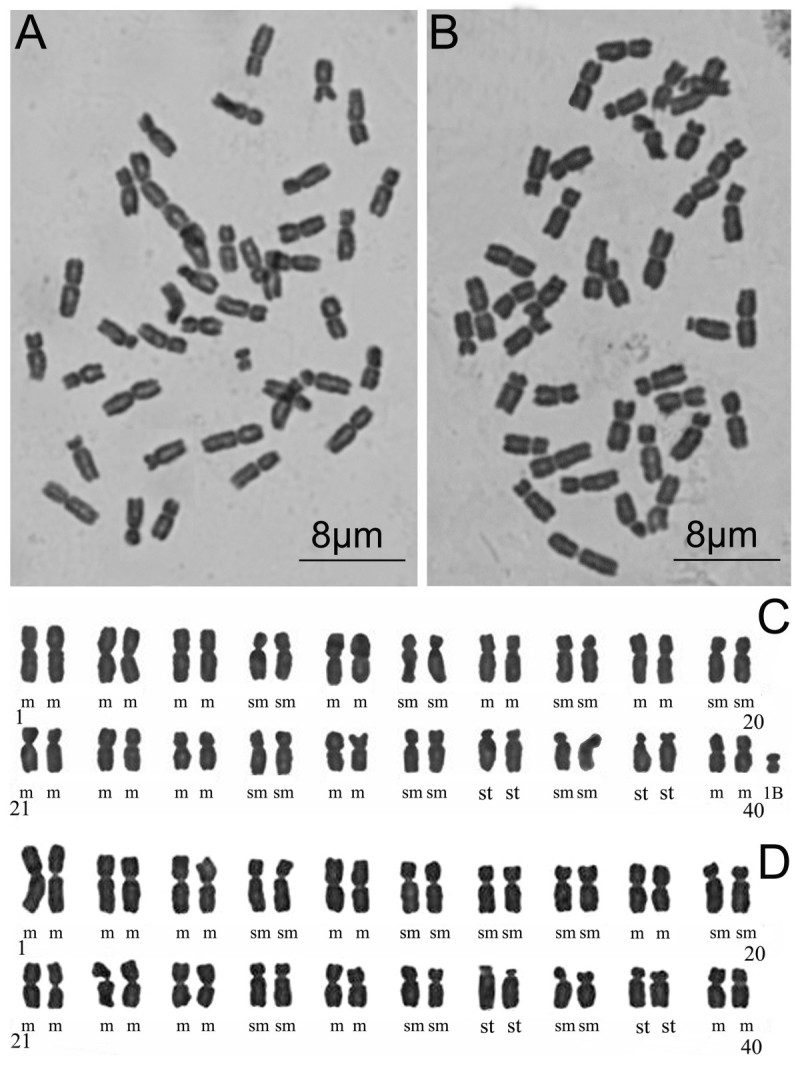
**Mitotic metaphase chromosomes (A, B) and karyotypes (C, D) of** ***Synotis xinningensis*** **(A, C) and** ***S. changiana*** **(B, D).** **A** and **C** from *Long-yuan Wang* &*Ming Tang 123* (HAST, IBSC) from the type locality; **B** and **D** from *Long-yuan Wang* &*Ming Tang 111* (HAST, IBSC) from Xing’an County, Guangxi, China.

The metaphase chromosomes of *Synotis xinningensis* were counted to be 2*n* = 40 + 0–1B (Figure 5A). According to the chromosome nomenclature of Levan et al. (1964), *S. xinningensis* had 22 median-centromeric (m), 14 submedian-centromeric (sm) and 4 subterminal-centromeric (st) chromosomes (Figure 5C), i.e. 2*n* = 40 = 22m + 14sm + 4st. In the six individuals examined, three were found to have one small B-chromosome in the metaphase cell. The metaphase chromosomes of *S. changiana* also were counted to be 2*n* = 40 (Figure 5B), including 20 m, 16 sm and 4 st (Figure 5D), i.e. 2*n* = 40 = 20m + 16sm + 4st. No B-chromosomes were observed in this species. In chromosome number and chromosome morphology, *Synotis xinningensis* is essentially the same as *S. changiana*.

The genus *Synotis* is poorly known cytologically, with reports of chromosome numbers for only three species. *Synotis alata* (Wall. ex DC.) C. Jeffrey & Y. L. Chen was reported from Indian material, under the name *Senecio alatus* Wall. ex DC., to have *n* = 20 (Mehra et al. 1965). The chromosomes of *S. rufinervis* (DC.) C. Jeffrey & Y. L. Chen were reported, also from Indian material and under the name *Senecio rufinervis* DC., to have *n* = 18 (Mehra and Remanandan 1975), *n* = 10 (Gupta and Gill 1981, 1989; Gupta et al. 2010), or *n* = 20 (Gupta and Gill 1989; Gupta et al. 2010). Liu et al. (2006) mentioned *S. lucorum* (Franch.) C. Jeffrey & Y. L. Chen, a plant endemic to northwestern Yunnan, China, to have 2*n* = 40. It should be noted that in that paper the plant was stated to have radiate capitula, but *S. lucorum* actually has discoid capitula (Jeffrey and Chen 1984; Chen 1999), so the material examined may have been misidentified or an error in observation occurred.

From the very limited chromosome data available, the most reliable basic chromosome number of *Synotis* appears to be *x* = 10, a basic number also characteristic of the genus *Senecio* L. as re-defined by Pelser et al. (2007). If this inference is correct, then both *Synotis xinningensis* and its putative closest relative, *S. changiana*, are tetraploid. More species of *Synotis* need to be examined cytologically to determine the variation pattern of the chromosomes and its systematic implications for the genus.

#### Notes

**Figure 6:**
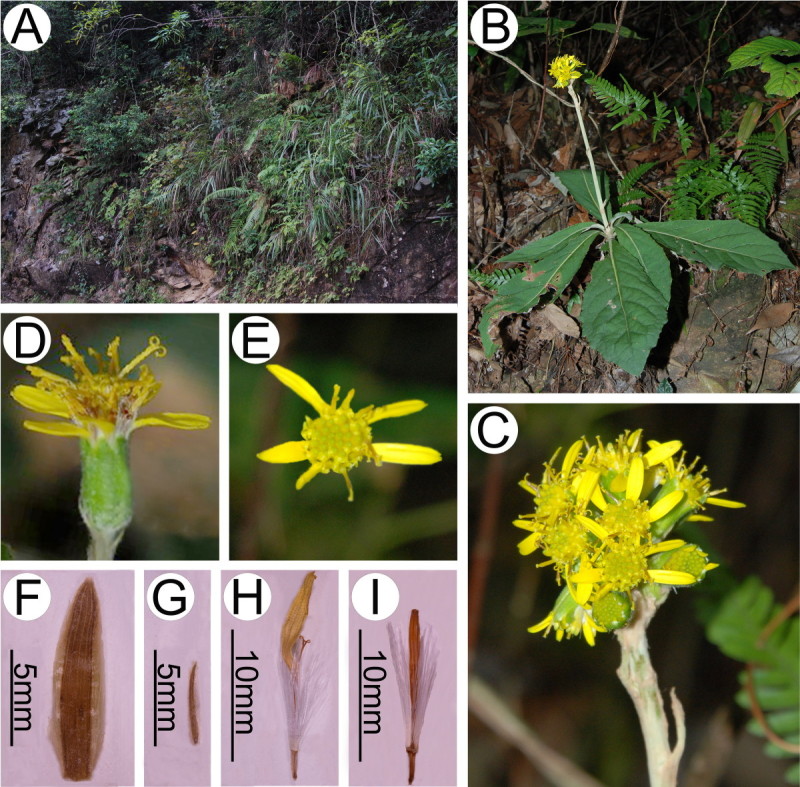
***Synotis changiana*** **. A**, Habitat; **B**, Habit; **C**, Synflorescence; **D**, Capitulum (side view); **E**, Capitulum (top view); **F**, Phyllary; **G**, Bract of calyculus; **H**, Ray floret; **I**, Disc floret. All from *Long-yuan Wang* &*Ming Tang 111* from Xing’an County, Guangxi, China (HAST, IBSC).

**Figure 7:**
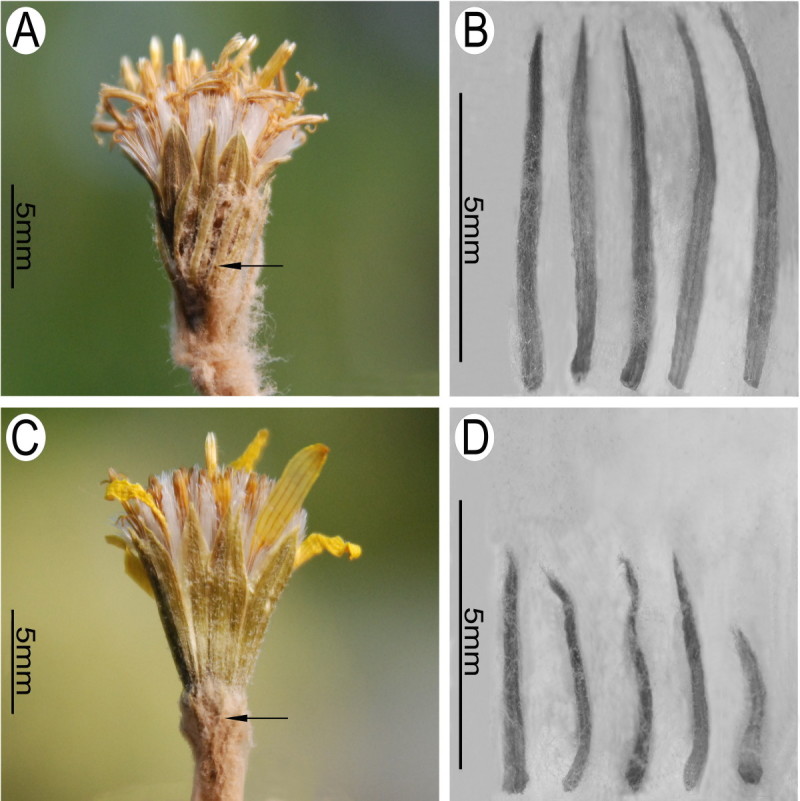
**Capitula (A, C) and bracts of calyculus (B, D) in** ***Synotis xinningensis*** **(A, B) and** ***S. changiana*** **(C, D), showing differences in length of bracts of calyculus (arrows).** **A** and **B** from *Long-yuan Wang* &*Ming Tang 123* (HAST, IBSC) from the type locality; **C** and **D** from *Long-yuan Wang* &*Ming Tang 111* (HAST, IBSC) from Xing’an County, Guangxi, China.

*Synotis xinningensis* is most similar to *S. changiana* (Figure 6), but readily distinguishable in the discoid capitula (vs. radiate) (Figures 1 and 2, 6 and 7), and in the bracts of the calyculus 9–10 (vs. 6–8), 6–7 mm long (vs. 3–4 mm) (Figure 7).

*Synotis xinningensis* is distributed in southwestern Hunan, and *S. changiana* in northeastern Guangxi, so their distribution ranges are adjacent to each other, only some 80 km apart (Figure 3). Both species prefer similar habitats, growing in open mixed forests at elevations between 450 and 1000 m above sea level.

Jeffrey and Chen (1984), and Chen (1999) divided *Synotis* into two well-marked sections, sect. *Synotis* and sect. *Atractylidifoliae* C. Jeffrey & Y. L. Chen; all but one of the species (*S. atractylidifolia*) fall within the former, which itself is divisible into five not very clearly differentiated series. *Synotis xinningensis*, with its herbaceous leaves rosulate or subrosulate at the base of inflorescence, the lower part of stem leafless at anthesis, and the inflorescence terminal, can be readily referred to ser. *Synotis*. In addition to *S. xinningensis*, series *Synotis* now includes 19 species (Chen et al. 2011; this study). Within series *Synotis*, only *S. xinningensis* and *S. changiana* have leaves that are always abaxially densely gray-white arachnoid-tomentose; all other species of series *Synotis* have leaves that are abaxially only sparsely arachnoid or puberulent, and often glabrescent (Jeffrey and Chen 1984; Chen 1999; Chen et al. 2011).

## Conclusions

*Synotis xinningensis* is most similar to *S. changiana*, but readily distinguishable by having discoid capitula and 9–10 longer bracts of calyculus. Both species belong to *Synotis* ser. *Synotis*.

## Authors’ original submitted files for images

Below are the links to the authors’ original submitted files for images. Authors’ original file for figure 1 Authors’ original file for figure 2 Authors’ original file for figure 3 Authors’ original file for figure 4 Authors’ original file for figure 5 Authors’ original file for figure 6 Authors’ original file for figure 7
