# Supplementary figures and images for: Synotis xinningensis (Asteraceae), a new species from Hunan, China
Source: Bot Stud. 2013 Aug 23;54:16. doi: 10.1186/1999-3110-54-16 (PMC5432852; doi:10.1186/1999-3110-54-16)

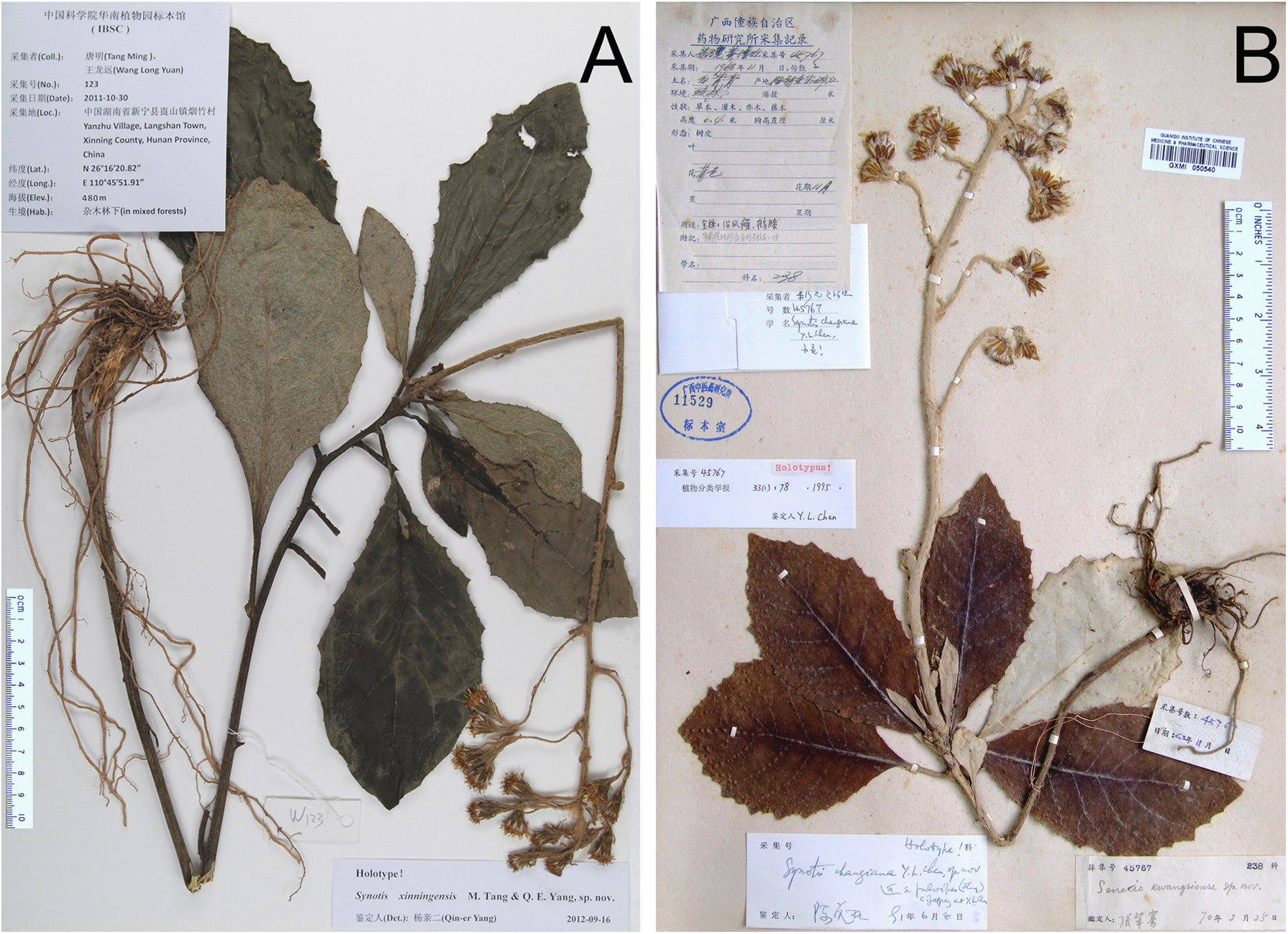

Supplement: Supplementary file 1 — Authors’ original file for figure 1 [file 40529_2012_15_MOESM1_ESM.tif]

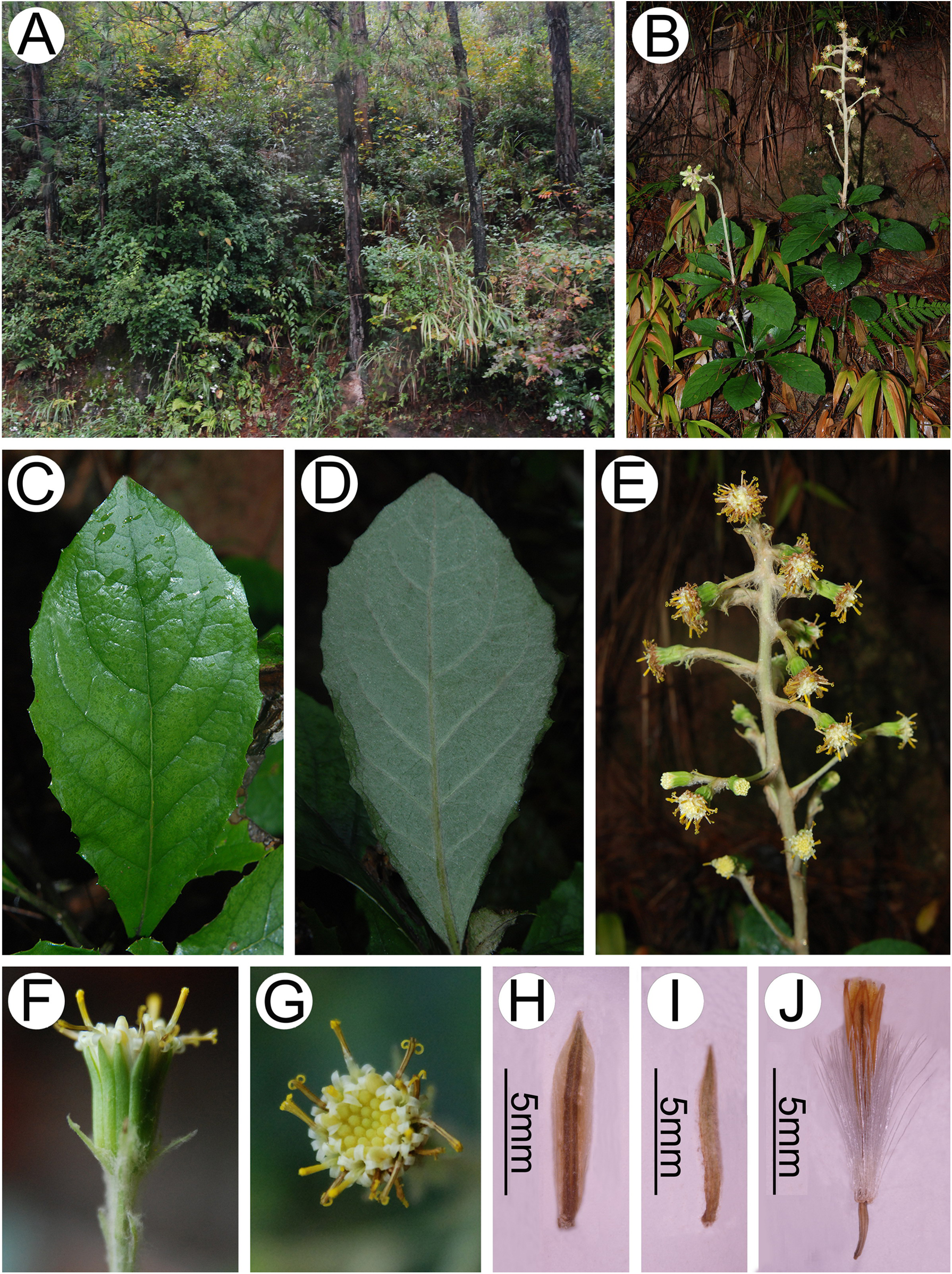

Supplement: Supplementary file 2 — Authors’ original file for figure 2 [file 40529_2012_15_MOESM2_ESM.tif]

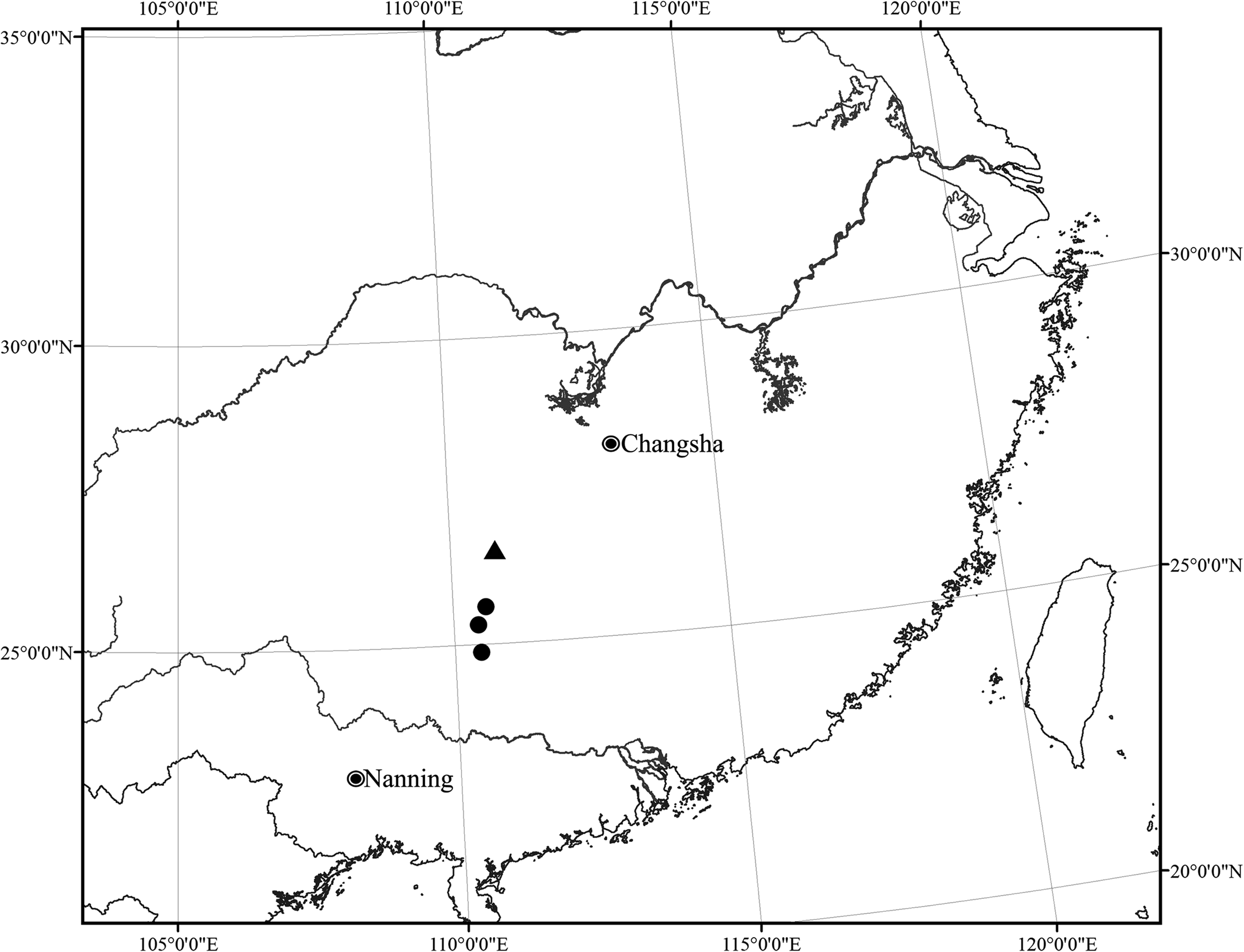

Supplement: Supplementary file 3 — Authors’ original file for figure 3 [file 40529_2012_15_MOESM3_ESM.tif]

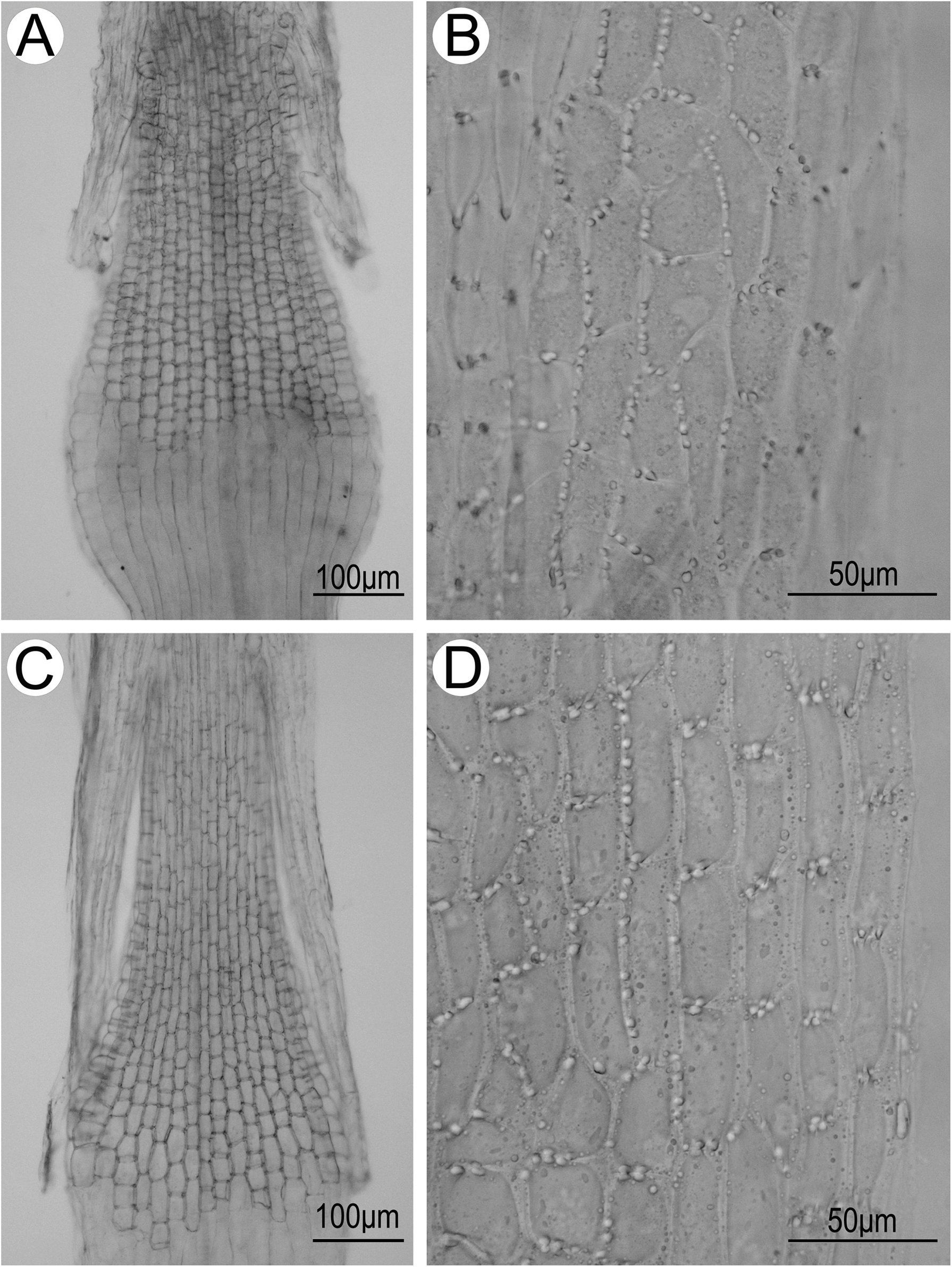

Supplement: Supplementary file 4 — Authors’ original file for figure 4 [file 40529_2012_15_MOESM4_ESM.tif]

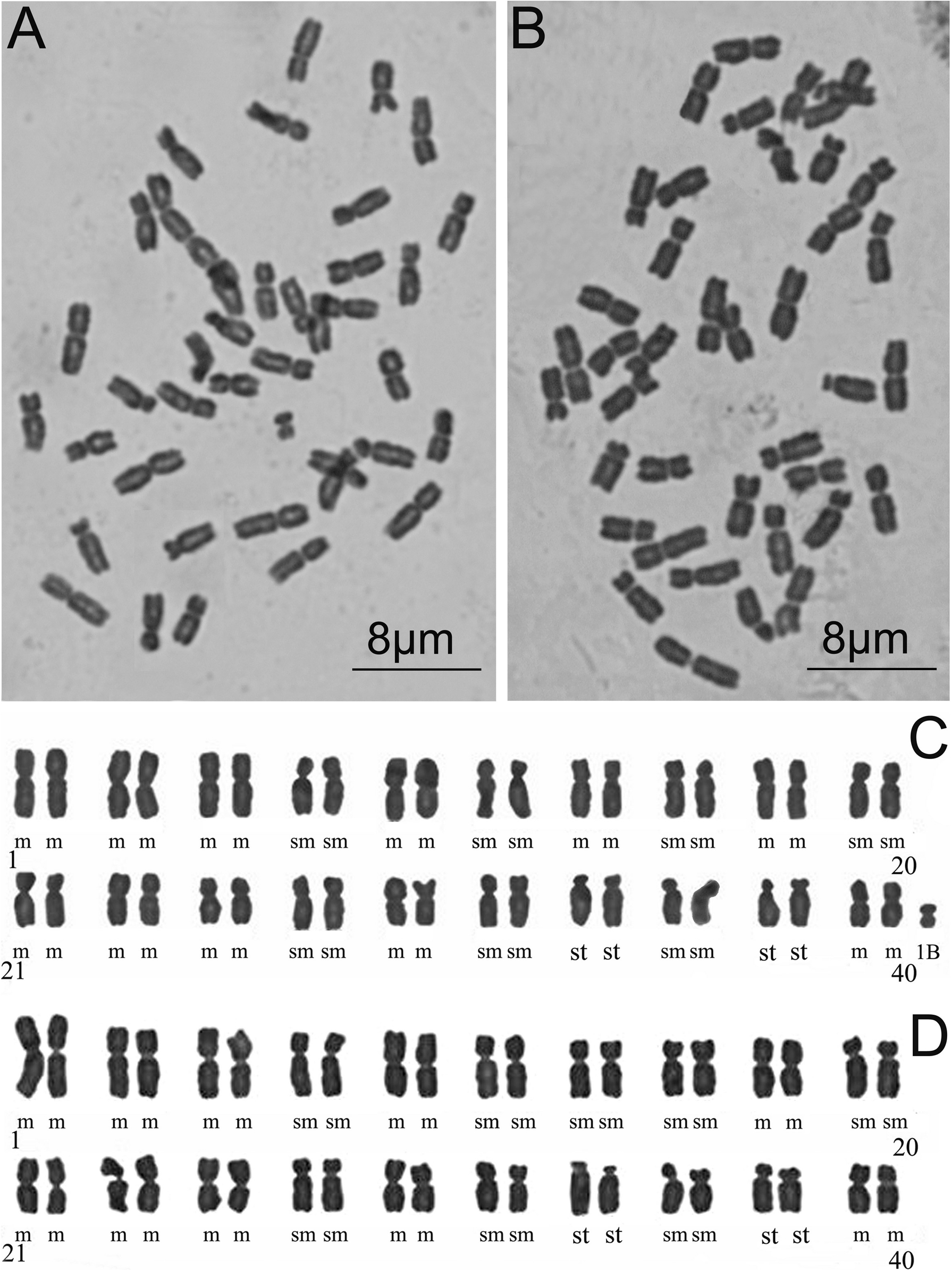

Supplement: Supplementary file 5 — Authors’ original file for figure 5 [file 40529_2012_15_MOESM5_ESM.tif]

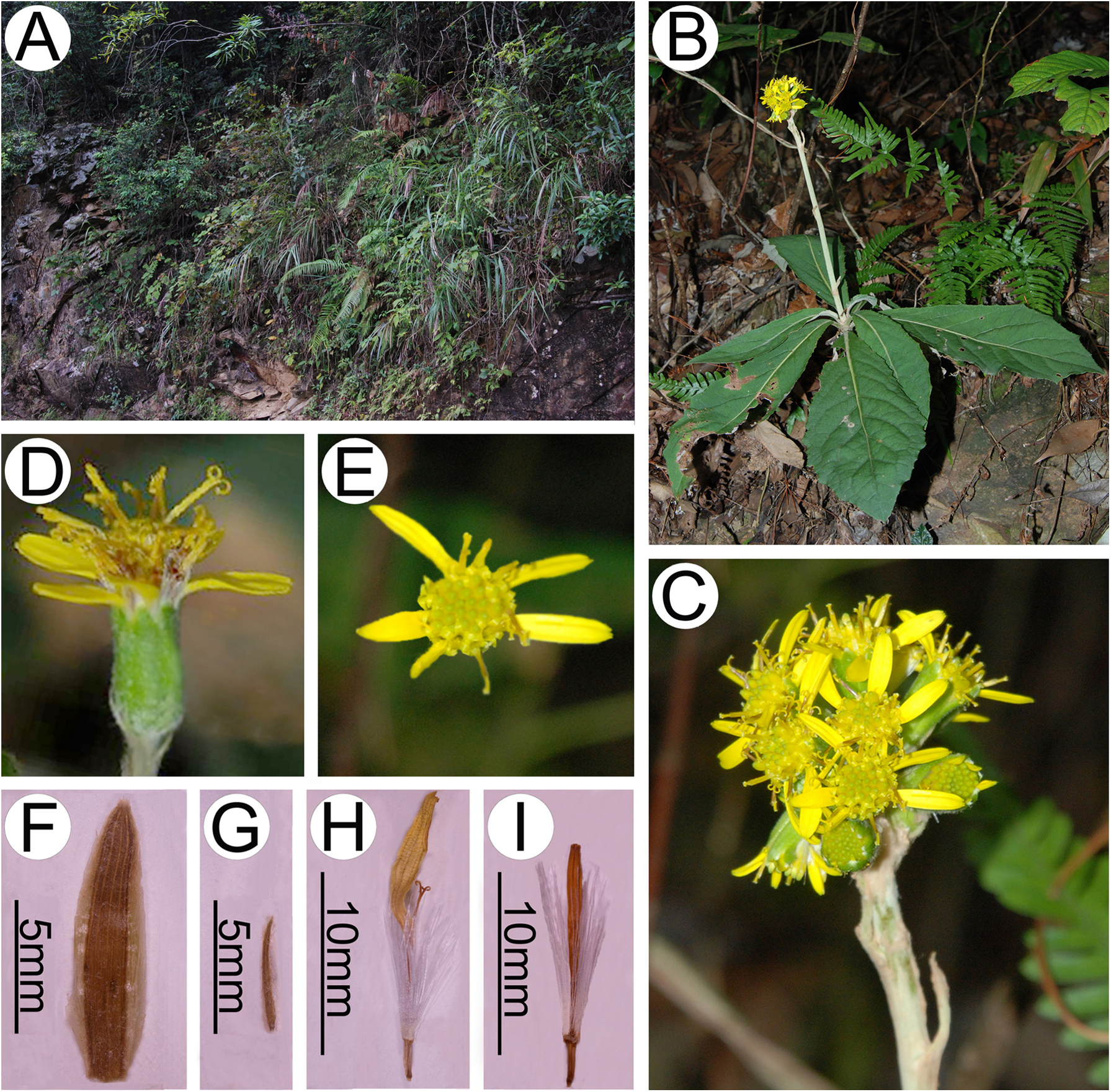

Supplement: Supplementary file 6 — Authors’ original file for figure 6 [file 40529_2012_15_MOESM6_ESM.tif]

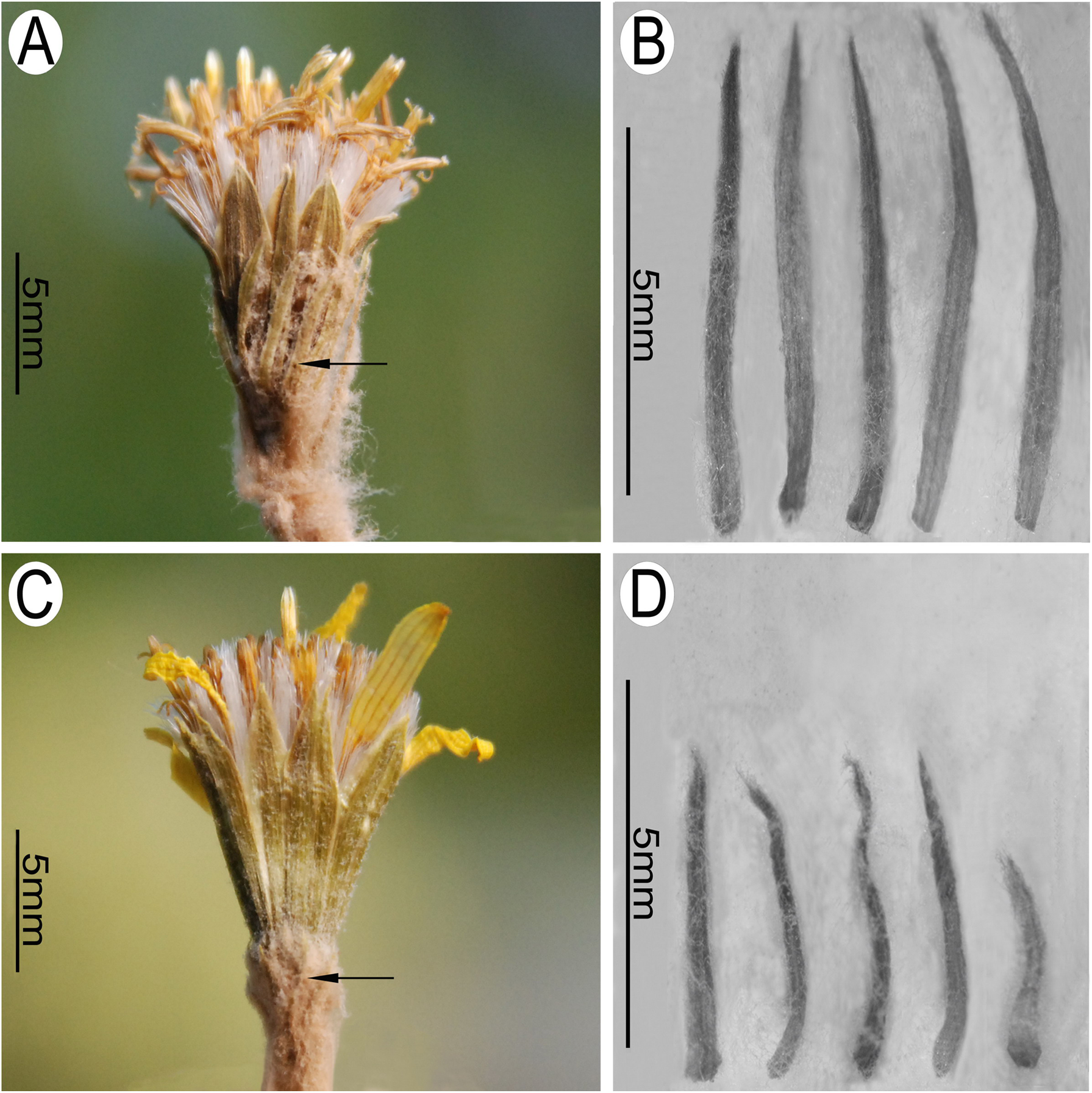

Supplement: Supplementary file 7 — Authors’ original file for figure 7 [file 40529_2012_15_MOESM7_ESM.tif]
